# Peripheral Retinal Vascular Patterns in Patients with Rhegmatogenous Retinal Detachment in Taiwan

**DOI:** 10.1371/journal.pone.0149176

**Published:** 2016-02-24

**Authors:** San-Ni Chen, Jiunn-Feng Hwang, Wen-Chuan Wu

**Affiliations:** 1 Department of Ophthalmology, Changhua Christian Hospital, Changhua City, Taiwan; 2 School of Medicine, Kaohsiung Medical University, Kaohsiung, Taiwan; 3 School of Medicine, Chung-Shan Medical University, Taichung, Taiwan; University of Sydney, AUSTRALIA

## Abstract

This is an observational study of fluorescein angiography (FA) in consecutive patients with rhegmatogenous retinal detachment (RRD) in Changhua Christian Hospital to investigate the peripheral retinal vascular patterns in those patients. All patients had their age, sex, axial length (AXL), and refraction status (RF) recorded. According to the findings in FA of the peripheral retina, the eyes were divided into 4 groups: in group 1, there was a ramified pattern of peripheral retinal vasculature with gradual tapering; in group 2, there was an abrupt ending of peripheral vasculature with peripheral non-perfusion; in group 3, there was a curving route of peripheral vasculature forming vascular arcades or anastomosis; and in group 4, the same as in group 3, but with one or more wedge-shaped avascular notches. Comparisons of age, sex, AXL, and RF, association of breaks with lattice degeneration and retinal non-perfusion, surgical procedures utilized, and mean numbers of operations were made among the four groups. Of the 73 eyes studied, there were 13 eyes (17.8%) in group 1, 3 eyes (4.1%) in group 2, 40 eyes (54.8%) in group 3 and 17 eyes (23.3%) in group 4. Significant differences in age, AXL and RF, and association of retinal breaks to non-perfusion were noted among the four groups. Patients in group 1 had older ages, while younger ages were noted in groups 3 and 4. Eyes in group 1 had the shortest average AXL and were least myopic in contrast to the eyes in groups 3 and 4. Association of retinal breaks and retinal non-perfusion was significantly higher in groups 2, 3 and 4 than in group 1. In conclusion, peripheral vascular anomalies are common in cases with RRD. Patients with peripheral non-perfusion tend to be younger, with longer axial length and have the breaks associated with retinal non-perfusion.

## Introduction

Rhegmatogenous retinal detachment (RRD) is a major cause of vision loss. The annual incidence of RRD ranges from 7.98 to 18.2 per 10^5^ person-years [1–4]. Myopia, trauma, previous cataract surgery, male sex, and congenital anomalies were reported as predisposed to the development of RRD [5–8]. Eyes with peripheral retinal vascular anomalies such as familial exudative vitreoretinopathy (FEVR)[9–12] and retinopathy of prematurity (ROP)[11–13] have also been reported to increase the chance of retinal breaks formation and the incidence of RRD. Since an avascular retina may render the retina to be more atrophic and easily torn by abnormal vitreoretinal adhesion, we thus suspect abnormal peripheral retinal non-perfusion may be more commonly observed in patients with RRD. In this study, we investigated the peripheral retinal vascular status in patients with RRD by using fluorescein angiography to see if peripheral retinal anomalies were present, and evaluated whether the anomalies were associated with age, sex, refractive status, axial length, retinal breaks formation, surgical procedures and times of operation.

## Materials and Methods

This is an observational study to evaluate the peripheral retinal vasculature by fluorescein angiographic (FA) in consecutive cases with RRD without previous history of surgical management for RRD in Changhua Christian Hospital, Taiwan from Jan 2011 to June 2011. The study was approved by the Institutional Review Boards of Changhua Christian Hospital. Exclusion criteria included the following: patients under the age of 18 years, history of prematurity, patients with dragging of the fovea in either eye, retinal vascular disease including occlusive retinal vasculitis, diabetic retinopathy, central and branch retinal vein occlusion, central and branch retinal artery occlusion, perforating ocular trauma history, macular hole associated RRD, previous ocular surgeries other than an uncomplicated cataract surgery, and systemic anomalies including Marfan syndrome and atopic dermatitis, which would increase the incidence of RRD, and severe proliferative vitreoretinopathy or bullous RD, which would interfere with the interpretation of FA.

All patients underwent color fundus photography and FA, which specifically covered the peripheral retina as far as possible. The color images were taken by a Canon fundus camera (Canon CR-2 Camera, Tokyo, Japan). The FA images were taken by HRA II (Heidelberg retinal angiography, Heidelberg, Germany) with a 55-degree lens. Patients were instructed to gaze upward, temporal-upper, temporal, temporal-lower, lower, nasal-lower, nasal, and nasal-upper to catch the image of peripheral fundus as far as possible. We recorded the vascular patterns of peripheral retina, whether the breaks were associated with non-perfusion of retina, the presence or absence of lattice degeneration and whether the location of lattice degeneration inside or outside the vascular zone. Other information including patients’ age, sex, axial length (AXL), refractive status, surgical procedures and times of operations were also recorded. The AXL was measured with a A-scan machine (A-2500, Sonomed, New Hyde Park, NY, USA) after the retina was attached. The refractive status of patients was also recorded. For patients with macula-on RRD, the refractive status was checked by the auto-refractometer (Canon-R-22, Tokyo, Japan) preoperatively. For patients with macula-off retinal detachment, since the original refractive status could not be checked by the auto-refractometer, and the refractive status would be changed by the surgical procedures (either with scleral buckle or vitrectomy), the refractive status was traced by taking the past history of refraction, or by measuring the refraction of the spectacles for far vision. Patients were divided into four groups according to the findings of FA as follows. Group 1 had a normal ramified pattern of peripheral retinal vasculature, peripheral avascular zone was within normal range (less than 1mm or 0.5 disc diameter). Group 2 had an abrupt ending of peripheral vasculature with terminal dilatation, losing the ramified vascular patterns and with wider peripheral non-perfusion. Neither circumferential route of the peripheral retinal vasculature, nor definite anastomosing vascular loop were noted. Group 3 had a curving route of peripheral vasculature forming an arcade or anastomosing vascular loop, and wide peripheral non-perfusion. Group 4 was the same as group 3, only with one or more avascular notches at the periphery. The assignment of groups was done by Dr. San-Ni Chen and Jiunn-Feng Hwang. If there were disagreements about the assignment of any patient between the two authors, the third author (Dr. Wen-Chuan Wu) would be asked to do the final assignment. The study adhered to the declaration of Helsinki. Informed consent was obtained from all subjects and the study protocol was approved by the Institutional Review Board of the Changhua Christian Hospital.

### Statistics

Fisher’s exact test was used to test the difference of sex distribution, presence of lattice degeneration and whether the breaks were associated with non-perfusion among the four groups, while the Chi-square test was used to test the difference between surgical procedures and the Kruskal-Wallis test was used for the calculation of the difference of age, axial length, refraction, number of breaks, and number of operations. The post-hoc test was used for the comparison of age, axial length, refraction and number of breaks between each group. All analyses were performed using R version 3.1.2 (The R Foundation for Statistical Computing, R Core Team, Vienna, Austria). A p value of less than 0.05 was considered to be statistically significant.

## Results

There were 73 eyes in seventy-three patients (42 males and 31 females) included in this study. All eyes had retina attached at final status. Twenty-five eyes had segmental scleral buckle, 43 eyes had vitrectomy and 5 eyes had combined vitrectomy and segmental scleral buckle. There were 13 eyes (17.8%) in group 1, 3 eyes (4.1%) in group 2, 40 eyes (54.8%) in group 3 and 17 eyes (23.3%) in group 4. No difference in the ratio of sex was noted among the four groups (p = 0.934, Table 1). Breaks associated with lattice degeneration were present in 59 eyes (79.7%), and no significant difference for whether the breaks were associated with lattice degeneration was noted between the four groups (Table 1, p = 0.252). For the association of breaks and retinal non-perfusion, there were 69.5% of lattice degeneration associated breaks, and 73.3% of non-lattice degeneration associated breaks related to retinal non-perfusion. A significant difference was noted among the four groups, eyes in groups 2, 3 and 4 but not 1 had retinal breaks highly associated with non-perfusion (Table 1, p<0.001). Significant difference was seen among the four groups for whether using scleral buckle alone (p = 0.002, Table 1). Eyes in group 2 tended not to receive buckle procedures alone to have the retina reattached.

**Table 1:** Comparison of the ratio of gender, whether the retinal breaks associated with lattice degeneration, and located within the non-perfusion area among the 4 groups.

	All (n = 73)	Group 1 (n = 13)	Group 2 (n = 3)	Group 3 (n = 40)	Group 4 (n = 17)	P-value
Ratio of male (%)	57.5	53.8	66.7	60.0	52.9	P = 0.934^a^
Breaks associated with LD (%)	79.5	69.2	100	75.0	94.1	P = 0.252^a^
Breaks located within NP (%)	71.2	23.1	100	80.0	82.4	P<0.001^a^
Surgical procedures with buckle alone	25	5	1	14	5	P = 0.002^b^

LD, lattice degeneration; NP, non-perfusion area; Y, yes; N: no.

^a^ P-value by Fisher’s exact test

^b^ P-value by Chi-square test

The mean age for all patients was 48.1±14.3 years (range 18–81 years). Significant difference of age was noted among the four groups of patients (p<0.001, Table 2). Patients in group 4 were the youngest and patients in group 1 had the oldest average age (41.7±11.1 years vs 59.8±10.6 years).The mean AXL was 25.87±1.75mm (range 22.7–30.4mm) for all, and significant differences between the four groups of eyes existed (p<0.001, Table 2). Eyes in group 4 had the longest AXL, and eyes in group 1 had the shortest AXL (26.73±1.61mm vs 24.02±0.96mm). The percentage of eyes with AXL less than 24 mm was 53.8%, 0%, 2.5%, 0% and 0% in groups 1, 2, 3, and 4 respectively. The percentage of eyes with AXL equal to or greater than 26.5mm was 0%, 0%, 37.5%, and 53.0% in groups 1, 2, 3, and 4 respectively. As for the refractive status, the mean refraction was -3.93±3.93D for all (range: -14.0D to +3.5D). Significant difference was noted between the four groups (Table 2, p<0.001). Eyes in group 1 were the least myopic (mean:-0.33±2.31 D) and eyes in group 4 were the most myopic (-7.03±3.03D). The mean number of operations was 1.19±0.54, without significant difference among the four groups (Table 2, p = 0.322).

**Table 2:** Comparison of age, axial length, refractive status and number of breaks between the 4 groups.

	All (n = 73)	Type 1 (n = 13)	Type 2 (n = 3)	Type 3 (n = 40)	Type 4 (n = 17)	P-value
Age (yr)	48.1±14.3	59.8±10.6	56.7±5.1	46.3±14.8	41.7±11.1	P<0.001
AL(mm)	25.87±1.75	24.02±0.96	25.21±0.86	26.16±1.66	26.73±1.16	P<0.001
Refraction (D)	-3.93±3.93	-0.33±2.31	-1.33±3.21	-3.88±3.66	-7.03±3.07	P<0.001
No. of breaks	2.04±1.32	1.92±1.12	2.67±1.15	2.08±1.47	1.94±1.14	P = 0.621
No. of operations	1.19±0.54	1.15±0.38	2.00±1.73	1.10±0.38	1.29±0.59	P = 0.322

Yr, years; AL, axial length; D,diopter; n, number of eyes.

For the intergroup comparison (Table 3), significant difference of age, and AXL was noted between group 1 and groups 3, and 4. Significant difference of RF was noted between group 1 and groups 3, 4, and between group 3 and group 4. Significant difference for the association of breaks and retinal non-perfusion was noted between group 1 and groups 2, 3 and 4.

**Table 3:** P value of Intergroup comparison between types of peripheral vascular patterns in age, axial length, refraction, and location of breaks.

	Age			Axl^a^			Ref^a^			Break		
	Type2	Type3	Type4	Type2	Type3	Type4	Type2	Type3	Type4	Type2	Type3	Type4
Type1	0.997	0.012	0.001	0.701	0.001	<0.001	0.995	0.022	<0.001	0.036	<0.001	0.002
Type2	-	0.486	0.160	-	0.767	0.501	-	0.555	0.065	-	1.000	1.000
Type3	-	-	0.402	-	-	0.750	-	-	0.045	-	-	1.000

Axl, Axial length; Ref, Refraction; Break, Break located within non-perfusion.

^a^ P-value by post-hoc test

^b^ P-value by Fisher’s exact test.

### FA findings of peripheral retina

#### Peripheral vascular changes in group 1

**Fig 1:**
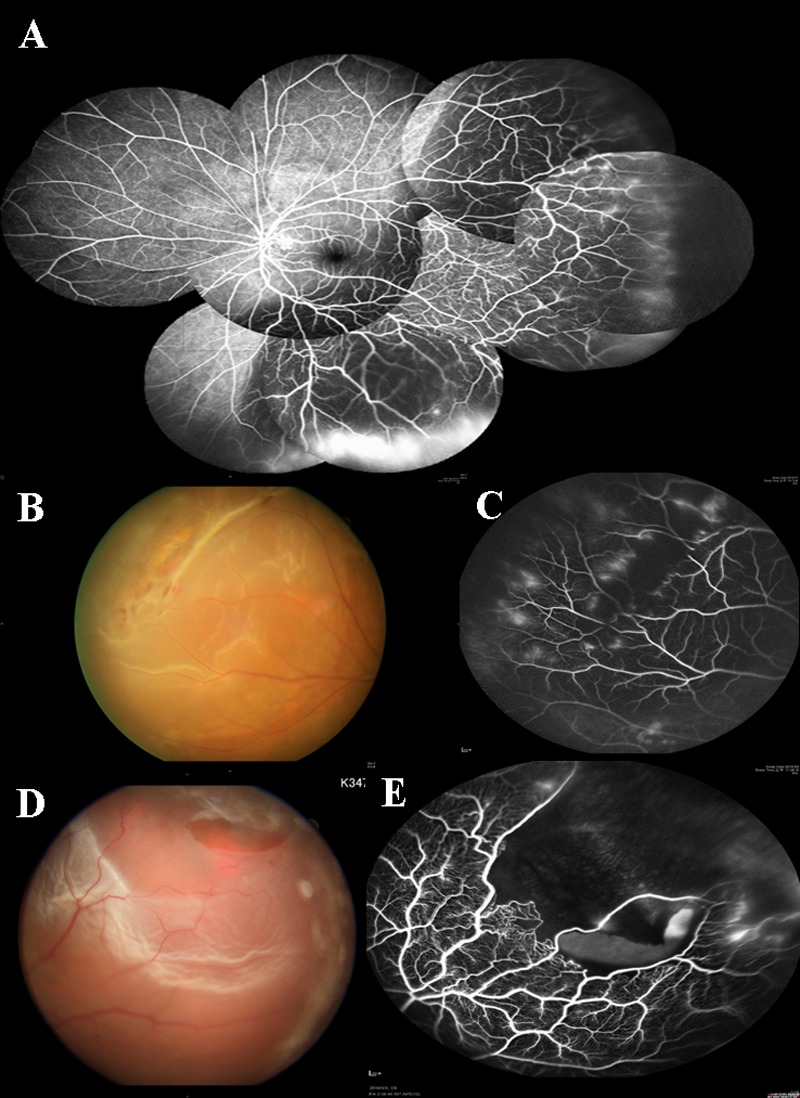
Color and FA pictures of group 1 eyes. (A) FA of an eye with group 1 retinal vasculature. Note there is a dichomatous ramified pattern of peripheral retinal vasculature with gradual tapering. Marked leakage is noted in the detached, peripheral retina. Area of peripheral retinal avascularity was noted, which was within 1mm in width. (B) Fundus photo of a lattice degeneration within the vascular zone in an eye with type 1 vascular pattern. (C) Fluorescein angiography of the lattice degeneration showing large vessels crossing over the lattice degeneration, with loss of the small vasculature within the area of lattice degeneration. Telangiectasia was noted at the peripheral retinal vasculature outside the area of lattice degeneration with normal dichomatous vascular pattern. Vascular leakage was noted at some parts of peripheral retinal vasculature and around the lattice degeneration. (D) Fundus photo of a lattice degeneration associated tear at the margin of vascular zone with type 1 vascular pattern. (E) Fluorescein angiography showing loss of small vasculature within the lattice degeneration and lack of vasculature peripheral to the lattice degeneration. Vascular anastomosis at inner margin of lattice degeneration and telangiectatic vessels within detached retina were also noted.

All eyes (13) in this group had a ramified pattern of peripheral retinal vasculature with gradual tapering (Fig 1A). The end of the retinal vasculature could be observed at the temporal side and the detached retina in all patients. Peripheral avascular area, which was within 1mm in width, was noted in all eyes. Vascular telangiectasia and leakage were observed at the area of the detached retina. Breaks associated with lattice degeneration was present in 9 of the 13 eyes; 8 of the 9 eyes had the lattice degeneration within the vascular area, though loss of capillaries and small vasculature were noted within the zone of lattice degeneration, leaving a pruning appearance of larger vessels transversing the lattice degeneration (Fig 1B and 1C), and 1 eye had the lattice degeneration situated at the margin of the vascular zone (Fig 1D and 1E). Of the 4 eyes without lattice degeneration, 2 eyes had the breaks in the vascular area, and 2 eyes had the breaks within the peripheral non-perfusion area.

#### Peripheral vascular changes in group 2

**Fig 2:**
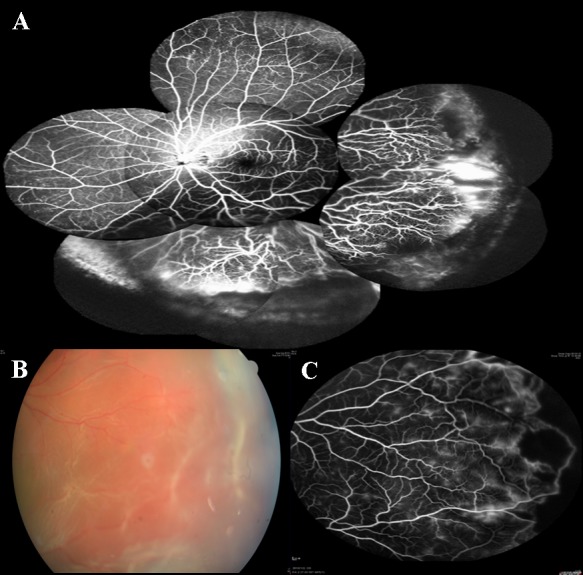
Color fundus and FA pictures of group 2 eyes (A) Fluorescein angiography of an eye with group 2 retinal vasculature. Note there is an abrupt cessation of temporal and lower peripheral retinal vasculature, leaving a wide area of peripheral non-perfusion. Marked leakage is noted at the temporal and lower detached retina. Some microaneurysms are noted in the nasal peripheral, attached retina. (B) Magnified color fundus showing lattice degeneration at temporal aspect in the same patient. (C) Fluorescein angiography at the same location showing the lattice degeneration situated just beyond the vascular cessation. Terminal dilatation, vascular tortuosity and telangiectatic vessels were also noted

All eyes (3) in this group had abrupt termination of some part of the peripheral retinal vasculatures with abnormally wide nonperfused peripheral retina. No circumferential route of vasculature or anastomosing vascular loops were noted (Fig 2A). Lattice degeneration was present in all 3 eyes, situated just beyond the abrupt termination of the retinal vasculature without obvious anastomosing vessels (Fig 2B and 2C). The other parts of the peripheral retinal vasculature, when visible, had a ramified pattern with gradual tapering. Telangiectasia and vascular leakage were observed at the detached retina and some also at the non-detached area.

#### Peripheral vascular changes in group 3

**Fig 3:**
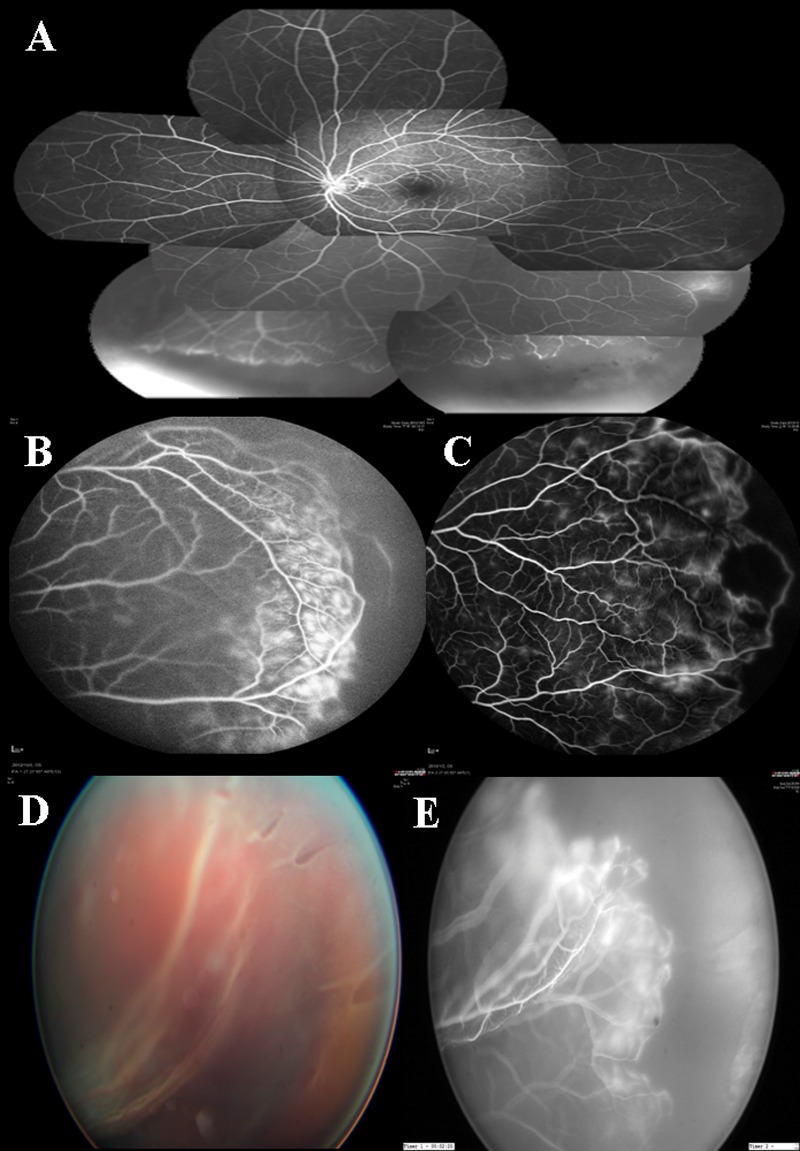
Color fundus and FA photos of group 3 eyes (A) Fluorescein angiography of an eye with group 3 retinal vasculature. Note there is anastomosis at the temporal and lower peripheral vasculature, with a wide non-perfusion at the temporal and lower aspect being noted. Vascular leakage is noted at some parts of the inferior and peripheral detached retina. (B) Another eye with type 3 vascular pattern showing circumferential route of peripheral vasculature, with peripheral non-perfusion, telangiectatic vessels and marked leakage. (C) Another eye with type 3 vascular pattern showing circumferential route and anastomosing vasculature forming a vascular loop with non-perfusion within the loop. Vascular leakage and telangiectasia were also noted. (D) Fundus photo of an eye with type 3 vascular pattern showing multiple tears without lattice degeneration. (E) Fluorescein angiography of the same patient showing that all the tears are in the peripheral non-perfused retina.

Eyes in this group (40 eyes) had at least some part of the peripheral retinal vasculature in a curving route without a ramified pattern (Fig 3A). Some of the peripheral vasculature formed an arc (Fig 3B), and some had end to end anastomosis of the peripheral retinal vasculature forming a closed loop with or without non-perfusion within the loop (Fig 3C). Lattice degeneration was present in 30 of the 40 eyes, of which 24 eyes had lattice degeneration within the peripheral avascular zone or at the outer margin of the vascular zone. In the 10 eyes without lattice degeneration, 9 eyes had the breaks in the non-perfused area, either in the peripheral avascular zone (Fig 3D and 3E), or in the non-perfusion area within the peripheral vascular loop. Telangiectasia and vascular leakage were observed in most eyes at the area of the detached retina.

#### Peripheral vascular changes in group 4

**Fig 4:**
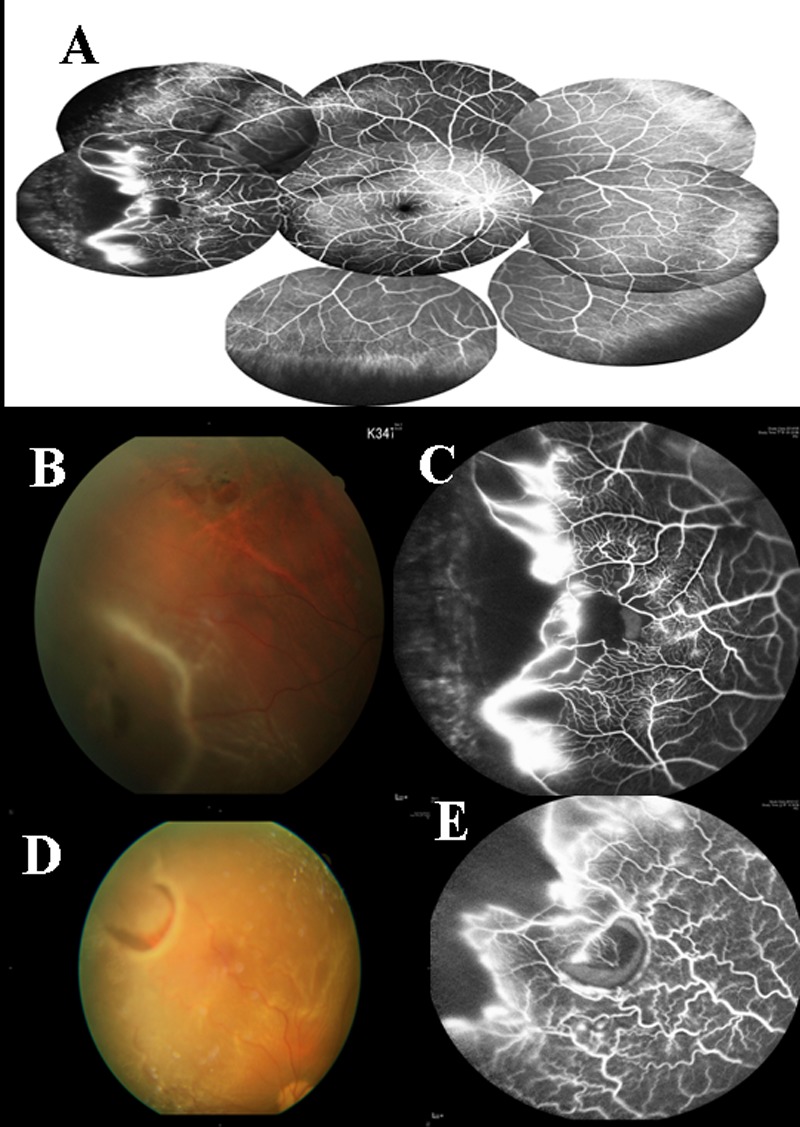
Color fundus and FA photos of group 4 eyes (A) Fluorescein angiography of group 4 vasculature. There is a V-shaped notch at peripheral retina, with peripheral anastomosing vasculature. Non-perfusion areas are noted inside the vascular arch. Wide area of peripheral non-perfusion is noted outside the temporal notch. Marked vascular leakage is noted at temporal anastomosing vessels. (B) Fundus photo of an eye with type 4 vascular pattern showing a tear at the temporal detached retina and 2 breaks associated with lattice degeneration at temporal upper attached retina. (C) Fluorescein angiography of the same eye, showing an avascular notch at temporal aspect and anastomosing vasculature. The tear is within the vascular loop, where the retina is non-perfused. (D) Fundus photo of a horse-shoe tear in an eye with type 4 vasculature. (E) Fluorescein angiography at the same location showing 2 V-shaped notches of the avascular zone. The tear is within a vascular loop, where the retina was partially perfused. Marked leakage was noted at the anastomosing vasculature.

All eyes (17) in this group had one or more V-shaped or trapezoid indentations of peripheral non-perfusion (Fig 4A). Anastomosis of the far peripheral vasculature was also observed at some areas in all eyes. Lattice degeneration was observed in 16 of the 17 eyes, with 4 eyes having the lattice degeneration within the vascular zone, and the other 12 eyes having lattice degeneration at the margin or outside the vascular zone. Beside lattice degeneration-associated tears, isolated tears often happen within the peripheral vascular loop which enclosed an ischemic or partially ischemic area (Fig 4B, 4C, 4D and 4E). Telangiectasia and vascular leakage at the detached retina was noted in all eyes.

## Discussion

Peripheral retinal vasculature in normal population generally manifests as a clear ramified pattern.^14^ Vascular anastomosis in the periphery is rare both in emmetropic (0%) and highly myopic eyes (2.6%) in the report of Kaneko Y et al.[14]. Microaneurysm and telangiectasia, which were noted in our cases, are also not rarely observed in normal subjects, but seldom associated with dye leakage.^14^ In normal populations, peripheral avascular retina was reported to be approximately 1mm in width in Asdourian and Goldberg’s study by using fluorescein angiography [15], and 0.5 disc diameter (about 0.75mm) in Rutnin and Schepens’ study by using ophthalmoloscopy [16]; while Kaneko Y et al reported that peripheral avascular retina was rare in emmetropic eyes (4.8%) and frequently observed in highly myopic eyes (AXL>26.5mm)[14]. The difference of presence of peripheral avascular retina between these reports may be due to the difficulty in seeing far periphery in some emmetropic eyes with attached retina even by using an ultra-widefield camera.

In our study, only eyes in group 1 had normal ramified pattern of peripheral vasculature. Surprisingly, avascularity of the far peripheral retina was observed in all eyes in this group; the explanation for which being that the detached retina makes peripheral retina more easily seen, even without ultra-widefield fundus camera. However, the peripheral retinal avascularity seems to be within the normal range of retinal non-perfusion described by Asdouriana and Goldberg [15–16], which is different to the findings of non-perfused peripheral retina in FA, which was wider in the other groups.

In group 2, abrupt ending, terminal dilatation and marked leakage of peripheral retinal vasculature, along with a wider peripheral non-perfusion were noted. The abrupt ending of peripheral retinal vasculature has also been described in cases of high myopia [14], CRVO [17], and FEVR [18]. In this group, lattice degeneration was noted just situated beyond the termination of vasculature. Whether the obliteration of the peripheral retinal vessels is a congenital abnormality that predisposes to the formation of lattice degeneration or is an acquired condition secondary to lattice degeneration is unknown.

Eyes in groups 3 and 4 were noted to have circumferential vasculature, which parallels ora serrata, and anastomosing vascular loops with wide peripheral non-perfusion. Circumferential vascular loops in the far periphery in histology has been described by Spitznas M et al. as a characteristic finding [19]. Despite this, vascular loops revealed by FA in groups 3 and 4 have seldom been observed in normal population. This may be explained by the fact that the anastomosing vascular loops become more prominent in these patients because of the retinal ischemia induced by retinal detachment, or because there is a wider peripheral non-perfusion area. The latter is a more likely reason, since vascular loops were not observed in group 1 eyes.

In addition to circumferential vasculature and anastomosing vascular loops, eyes in group 4 had one or more vascular notching, generally at the temporal aspect. Temporal vascular notching has been reported in patients with FEVR [18]. Since FEVR is a rare disease, and all these cases in group 4 had no obvious foveal dragging in both eyes, we are not sure whether these cases have the notching inborn or acquired and could be diagnosed as FEVR. It is interesting to note that patients in this group had only minor longer mean axial length, but were significantly more myopic than patients in group 3 (Table 3). Whether there is clinical significance is still unknown.

In addition to the common presence of abnormal peripheral non-perfusion in our series, we also noted that the development of the retinal breaks was closely associated with retinal non-perfusion. In those eyes with lattice degeneration, most of them had the lattice degeneration located within the peripheral non-perfusion area or at the outer margin of the vascular zone. Even in those eyes had the lattice degeneration situated within the vascular zone, the vasculature within the lattice degeneration was sclerotic and devoid of capillaries. Besides, for the non-lattice degeneration associated retinal breaks, most of them were located within the avascular area, either in the peripheral avascular zone, or within the avascular area in the peripheral vascular loop. Avascularity of the peripheral retina may render the retina ischemic and atrophic with the easy formation of breaks after traction. Furthermore, astrocytes were absent in the avascular retina [20]. It was reported that astrocytes and endothelial cells are interdependent cells, both of which are absent from the peripheral retinal avascular margin and foveal avascular zone [21]. Since astrocytes were important in maintaining the tensile strength of retina, lack of astrocytes in the peripheral avascular retina may make the retina weaker. Besides, astrocytes offer important support for the retinal vasculature and together with microglial cells, contribute to the perivascular glial limitans [22–23]. Thus, absence of astrocytes at the margin of vascular zone, may explain microaneurysms formations and the marked leakage noted in most of our patients at the peripheral retina.

We also noted that patients with abnormal peripheral non-perfusion, especially eyes in group 3 and group 4, tended to have longer axial length, be more myopic, and younger in age. Only less than 4 percent of patients in these 2 groups had an axial length less than 24mm. Myopia is an important risk factor for rhegmatogenous retinal detachment, and its importance as a risk factor is even higher in the younger age groups [5–7]. In the study of Kaneko et al [14], the area of avascularity of the far peripheral retina was noted in 82.6% of high myopic eyes with an axial length greater than 26.5mm (mean 28.5+- 1.5mm) and 4.8% in emmetropic subjects. The patterns of peripheral non-perfusion in our patients of types 3 and 4 are similar to those reported in the high myopic eyes. The differences are that in our study, though most eyes in groups 3 and 4 were only moderately myopic, 100% of them had abnormal peripheral non-perfusion, and almost all eyes had anastomosing vessels, in contrast to the low rates of anastomosing vessels reported by Kaneko et al. Though the peripheral vascular patterns in moderate myopic eyes (axial length less than 26.5mm) without retinal detachment are still known, it is reasonable to speculate the chance of peripheral non-perfusion is less than that in the high myopic, but more than that in the emmetropic eyes. The patients in groups 3 and 4 might be more vulnerable to the peripheral vascular changes secondary to axial elongation, which are supposed to be more often observed in high myopic eyes.

This finding may remind us about cautious use of laser to barricade the breaks in young, myopic patients, since there is a high chance that the retina around the breaks is avascular and atrophic, and lasers with strong energy may induce iatrogenic or atrophic holes around the laser spots. This may also indicate a cautious use of pneumatic retinopexy for repairing retinal detachment in those patients, since this procedure does not relieve vitreous traction permanently, but leaves an even more atrophic cryo-or laser- retinopexy scar on the already thin, avascular retina, which may increase the chance of new breaks formation around the scar in the future.

The pathogenesis of peripheral non-perfusion in our patients is unknown. Since most of our patients with peripheral nonperfusion tended to be myopic and have a longer axial length, the elongated eyeball might be a contributing factor. As the eye elongates, the original peripheral avascular retina may stretch to meet the elongation. Another possibility is that these patients may have been born with abnormal peripheral retinal vascular development. Since the peripheral retina may be associated with emmetropization and normal eye growth [24], a less vascularized peripheral retina, such as that in ROP, may predispose patients to abnormal eye growth and myopia development [25]. Moreover, it is also possible that the peripheral retinal vasculature regressed secondary to the thinner peripheral retina with an increased oxygen level from the choroid, as suggested by Kaneko Y et al[14].

One thing to be noted is that almost all our patients were Chinese in ethnicity. As myopia is much more prevalent in Chinese populations, the refractive status in patients of RRD in other ethnic groups may differ. It is possible that eyes with RRD in different ethnic groups, in which myopia is not that prevalent, would not have such a high chance of abnormal peripheral non-perfusion.

## Conclusion

In summary, peripheral retinal vascular anomalies are frequently observed in Chinese patients with RRD, especially in younger age groups, and in eyes with myopia. The distribution of lattice degeneration and breaks was also associated with the retinal non-perfusion in most of our cases. Peripheral vascular anomalies may be an important contributing factor for the formation of RRD. The weak points of the study are the insufficient number of cases (especially as there are only 3 cases in group 2, from which we could not draw any valid conclusions), lack of a control group, and not using ultra-widefield angiography in the evaluation of peripheral vasculature, though with the help of instructing patients gazing at different directions and with the tilt of the fundus camera, we could still asses the vascular patterns of the peripheral retina. Besides, almost all patients in this study were Chinese; the results may only represent the clinical characteristics of Chinese patients. Further studies with ultra- widefield angiography with a control group and cross-ethnic studies may better establish the peripheral vascular anomalies in patients with RRD.

## Supporting Information

S1 File Supplementary Excel file with raw data. (XLSX)

S2 File STROBE Statement—checklist of items that should be included in reports of observational studies. (DOCX)
