# Supplementary material for: Peripheral Retinal Vascular Patterns in Patients with Rhegmatogenous Retinal Detachment in Taiwan
Source: PLoS One. 2016 Feb 24;11(2):e0149176. doi: 10.1371/journal.pone.0149176 (PMC4766194; doi:10.1371/journal.pone.0149176)
Supplement: S2 File — (DOCX) [file pone.0149176.s002.docx]

STROBE Statement—checklist of items that should be included in reports of observational studies

|  | Item No. | Recommendation | Page  No. | Relevant text from manuscript |
| --- | --- | --- | --- | --- |
| **Title and abstract** | 1 | (*a*) Indicate the study’s design with a commonly used term in the title or the abstract | 2 | This is an observational study of fluorescein angiography (FA) in consecutive patients with rhegmatogenous retinal detachment (RRD) in Changhua Christian Hospital to investigate the peripheral retinal vascular patterns in those patients. |
|  |  | (*b*) Provide in the abstract an informative and balanced summary of what was done and what was found | 2 | peripheral vascular anomalies are common in cases with RRD. Patients with peripheral non-perfusion tend to be younger, with longer axial length and have the breaks associated with retinal non-perfusion |
| Introduction | | | |  |
| Background/rationale | 2 | Explain the scientific background and rationale for the investigation being reported | 3 | Eyes with peripheral retinal vascular anomalies such as familial exudative vitreoretinopathy (FEVR)[9-12] and retinopathy of prematurity (ROP)[11-13] have also been reported to increase the chance of retinal breaks formation and the incidence of RRD. |
| Objectives | 3 | State specific objectives, including any prespecified hypotheses | 3 | Since an avascular retina may render the retina to be more atrophic and easily torn by abnormal vitreoretinal adhesion, we thus suspect abnormal peripheral retinal nonperfusion may be more commonly observed in patients with RRD. |
| Methods | | | |  |
| Study design | 4 | Present key elements of study design early in the paper | 3, | This is an observational study |
| Setting | 5 | Describe the setting, locations, and relevant dates, including periods of recruitment, exposure, follow-up, and data collection | 3 | Changhua Christian Hospital, Taiwan, from Jan 2011 to June 2011. |
| Participants | 6 | (*a*) *Cohort study*—Give the eligibility criteria, and the sources and methods of selection of participants. Describe methods of follow-up  *Case-control study*—Give the eligibility criteria, and the sources and methods of case ascertainment and control selection. Give the rationale for the choice of cases and controls  *Cross-sectional study*—Give the eligibility criteria, and the sources and methods of selection of participants | 3 | consecutive cases with RRD without previous history of surgical management for RRD |
|  |  | (*b*) *Cohort study*—For matched studies, give matching criteria and number of exposed and unexposed  *Case-control study*—For matched studies, give matching criteria and the number of controls per case |  |  |
| Variables | 7 | Clearly define all outcomes, exposures, predictors, potential confounders, and effect modifiers. Give diagnostic criteria, if applicable |  |  |
| Data sources/ measurement | 8* | For each variable of interest, give sources of data and details of methods of assessment (measurement). Describe comparability of assessment methods if there is more than one group |  |  |
| Bias | 9 | Describe any efforts to address potential sources of bias | 4 | The assignment of groups was done by Dr. San-Ni Chen and Jiunn-Feng Hwang. If there were disagreements about the assignment of any patient between the two authors, the third author (Dr. Wen-Chuan Wu) would be asked to do the final assignment. |
| Study size | 10 | Explain how the study size was arrived at |  |  |

Continued on next page

| Quantitative variables | 11 | Explain how quantitative variables were handled in the analyses. If applicable, describe which groupings were chosen and why | 4 | Other information including patients’ age, sex, axial length (AXL) , refractive status, surgical procedures and times of operations were also recorded. The AXL was measured with a A-scan machine (A-2500, Sonomed, New Hyde Park, NY, USA) after the retina was attached. The refractive status of patients was also recorded. For patients with macula-on RRD, the refractive status was checked by the auto-refractometer (Canon-R-22, Tokyo, Japan) preoperatively. For patients with macula-off retinal detachment, since the original refractive status could not be checked by the auto-refractometer, and the refractive status would be changed by the surgical procedures (either with scleral buckle or vitrectomy), the refractive status was traced by taking the past history of refraction, or by measuring the refraction of the spectacles for far vision. |
| --- | --- | --- | --- | --- |
| Statistical methods | 12 | (*a*) Describe all statistical methods, including those used to control for confounding |  |  |
|  |  | (*b*) Describe any methods used to examine subgroups and interactions |  |  |
|  |  | (*c*) Explain how missing data were addressed |  |  |
|  |  | (*d*) *Cohort study*—If applicable, explain how loss to follow-up was addressed  *Case-control study*—If applicable, explain how matching of cases and controls was addressed  *Cross-sectional study*—If applicable, describe analytical methods taking account of sampling strategy | 4,5 | Fisher’s exact test was used to test the difference of sex distribution, presence of lattice degeneration and whether the breaks were associated with non-perfusion among the four groups, while the Chi-square test was used to test the difference between surgical procedures and the Kruskal-Wallis test was used for the calculation of the difference of age, axial length, refraction, number of breaks, and number of operations. The post-hoc test was used for the comparison of age, axial length, refraction and number of breaks between each group. All analyses were performed using R version 3.1.2 Copyright 2014 (The R Foundation for Statistical Computing, R Core Team, Vienna, Austria). A p value of less than 0.05 was considered to be statistically significant. |
|  |  | (*e*) Describe any sensitivity analyses |  |  |
| Results | | | | |
| Participants | 13* | (a) Report numbers of individuals at each stage of study—eg numbers potentially eligible, examined for eligibility, confirmed eligible, included in the study, completing follow-up, and analysed | 5 | There were 73 eyes in seventy-three patients (42 males and 31 females) included in this study. |
|  |  | (b) Give reasons for non-participation at each stage |  |  |
|  |  | (c) Consider use of a flow diagram |  |  |
| Descriptive data | 14* | (a) Give characteristics of study participants (eg demographic, clinical, social) and information on exposures and potential confounders | 3 | This is an observational study to evaluate the peripheral retinal vasculature by fluorescein angiographic (FA) in consecutive cases with RRD without previous history of surgical management for RRD |
|  |  | (b) Indicate number of participants with missing data for each variable of interest |  |  |
|  |  | (c) *Cohort study*—Summarise follow-up time (eg, average and total amount) |  |  |
| Outcome data | 15* | *Cohort study*—Report numbers of outcome events or summary measures over time |  |  |
|  |  | *Case-control study—*Report numbers in each exposure category, or summary measures of exposure |  |  |
|  |  | *Cross-sectional study—*Report numbers of outcome events or summary measures | *5* | There were 73 eyes in seventy-three patients (42 males and 31 females) included in this study. There were 13 eyes (17.8%) in group 1, 3 eyes (4.1%) in group 2, 40 eyes (54.8%) in group 3 and 17 eyes (23.3%) in group 4. for whether using scleral buckle alone (p=0.002, Table 1). |
| Main results | 16 | (*a*) Give unadjusted estimates and, if applicable, confounder-adjusted estimates and their precision (eg, 95% confidence interval). Make clear which confounders were adjusted for and why they were included | 5 |  |
|  |  | (*b*) Report category boundaries when continuous variables were categorized |  |  |
|  |  | (*c*) If relevant, consider translating estimates of relative risk into absolute risk for a meaningful time period |  |  |

Continued on next page

| Other analyses | 17 | Report other analyses done—eg analyses of subgroups and interactions, and sensitivity analyses | 5 | No difference in the ratio of sex was noted among the four groups (p=0.934, Table 1). Breaks associated with lattice degeneration were present in 59 eyes (79.7%), and no significant difference for whether the breaks were associated with lattice degeneration was noted between the four groups (Table 1, p=0.252 ) . For the association of breaks and retinal non-perfusion, there were 69.5% of lattice degeneration associated breaks, and 73.3% of non-lattice degeneration associated breaks related to retinal non-perfusion. A significant difference was noted among the four groups, eyes in groups 2, 3 and 4 but not 1 had retinal breaks highly associated with non-perfusion (Table 1, p<0.001). Significant difference was seen among the four groups |
| --- | --- | --- | --- | --- |
| Discussion | | | | |
| Key results | 18 | Summarise key results with reference to study objectives | 13 | In summary, peripheral retinal vascular anomalies are frequently observed in Chinese patients with RRD, especially in younger age groups, and in eyes with myopia. The distribution of lattice degeneration and breaks was also associated with the retinal non-perfusion in most of our cases. Peripheral vascular anomalies may be an important contributing factor for the formation of RRD. |
| Limitations | 19 | Discuss limitations of the study, taking into account sources of potential bias or imprecision. Discuss both direction and magnitude of any potential bias | 13 | The weak points of the study are the insufficient number of cases (especially as there are only 3 cases in group 2, from which we could not draw any valid conclusions), lack of a control group, and not using ultra wide-field angiography in the evaluation of peripheral vasculature, though with the help of instructing patients gazing at different directions and with the tilt of the fundus camera, we could still asses the vascular patterns of the peripheral retina. Besides, almost all patients in this study were Chinese; the results may only represent the clinical characteristics of Chinese patients. |
| Interpretation | 20 | Give a cautious overall interpretation of results considering objectives, limitations, multiplicity of analyses, results from similar studies, and other relevant evidence | 13 | One thing to be noted is that almost all our patients were Chinese in ethnicity. As myopia is much more prevalent in Chinese populations, the refractive status in patients of RRD in other ethnic groups may differ. It is possible that eyes with RRD in different ethnic groups, in which myopia is not that prevalent, would not have such a high chance of abnormal peripheral nonperfusion. |
| Generalisability | 21 | Discuss the generalisability (external validity) of the study results |  |  |
| Other information | |  | | |
| Funding | 22 | Give the source of funding and the role of the funders for the present study and, if applicable, for the original study on which the present article is based |  | There is no funding support of this study. |

*Give information separately for cases and controls in case-control studies and, if applicable, for exposed and unexposed groups in cohort and cross-sectional studies.

**Note:** An Explanation and Elaboration article discusses each checklist item and gives methodological background and published examples of transparent reporting. The STROBE checklist is best used in conjunction with this article (freely available on the Web sites of PLoS Medicine at http://www.plosmedicine.org/, Annals of Internal Medicine at http://www.annals.org/, and Epidemiology at http://www.epidem.com/). Information on the STROBE Initiative is available at www.strobe-statement.org.
